# Activating knowledge-based practice through healthcare leadership development: insights from the second study of a broader action research project

**DOI:** 10.1186/s12913-026-14243-5

**Published:** 2026-02-25

**Authors:** Rita Solbakken, Trude Anita Hartviksen, Lars Strauman, Inger-Lise Magnussen

**Affiliations:** 1 Faculty of Nursing and Health Sciences, Nord University, Bodø, Norway; 2 Vestvågøy Municipality, Leknes, Norway; 3 Center for Care Sciences, North, UiT, The Arctic University of Norway, PO Box 6050, Langnes, Tromsø 9037 Norway; 4 Lofotleger AS, Leknes, Norway; 5 Faculty of Nursing and Health Sciences, Nord University, Stokmarknes, Norway

**Keywords:** Leadership development, Appreciative inquiry, Action research, Participatory research, Transformative learning, Municipal healthcare

## Abstract

**Background:**

The increasing complexity of healthcare services underscores the urgent need for competent and resilient leadership. Yet research on leadership development in healthcare remains limited, with notable gaps across theoretical frameworks and organizational contexts. This study aimed to explore how participating leaders experienced pedagogical and relational principles, used in workshops during the development of a continuous leadership program in rural municipal healthcare.

**Methods:**

This hermeneutic study is the second study of a broader action research project, conducted in a rural municipality and guided by an appreciative approach. Forty-one healthcare leaders from three leadership levels participated. Data was collected through 16 focus group interviews, participatory observations, and online surveys, and analyzed using Braun and Clarke’s reflexive thematic analysis.

**Results:**

The analysis identified one overarching theme: Activating a knowledge-based leadership community, supported by two interrelated themes: (1) Genuine involvement grounded in appreciative processes, (2) Facilitated interpersonal and professional knowledge development.

**Conclusions:**

This study demonstrates that activating knowledge-based leadership development in rural municipalities can be strengthened through the conscious choice of relational and participatory pedagogies. Our main contribution is a model of how pedagogical and relational principles—such as appreciation, involvement, and psychological safety—can be operationalized to build leadership capacity in municipal healthcare services. Leadership development appears most effective when embedded in shared experiences, supported by competent facilitators, and anchored in safe and appreciative environments. Within this context, psychological safety emerged as both a condition for, and a driver of, learning, interpersonal connection, and cultural change. The findings further highlighted the importance of continuity, organizational anchoring, and inclusive practices to sustain leadership development over time.

**Supplementary Information:**

The online version contains supplementary material available at 10.1186/s12913-026-14243-5.

## Background

### Healthcare leadership

Leadership is recognized as a critical factor in shaping organizational culture and ensuring high-quality care in healthcare services [1]. Healthcare leadership involves guiding teams and systems in ways that promote patient safety, quality improvement, and organizational learning. Over the past decades, leadership thinking has shifted away from hierarchical, top-down approaches toward models that acknowledge complexity, non-linearity, and adaptive processes [2]. For example, a participatory appreciative project showed how home-care leaders must balance ethical tensions between human needs and organizational economics, identifying the importance of value-based leadership grounded in ethical principles [3].

Cummings et al. [4] stated that relational leadership perspectives—such as transformational, resonant, and authentic leadership—have gained prominence due to their relevance in complex and high-demand environments. Transformational leadership is associated with increased job satisfaction, motivation, and teamwork [4]. Further, Singh et al. [5] argued that healthcare leadership requires a dynamic and adaptable approach that integrates multiple theories to address contextual challenges. Tailored leadership approaches are shown to improve both patient care and organizational functioning [1, 5].

Solbakken et al. [6] suggested that being a nurse leader entails relationship building, emphasizing the leaders’ need for integration in teams on their own units, with leaders at other units, and with leaders across the organization. However, leaders frequently face significant challenges, including limited human-resource competence [7], insufficient managerial support, a lack of learning environments, insufficient time for improvement efforts, a need for psychological safety [5] and established leadership networks for personal.

and professional support [8]. The leader’s wellbeing, proactiveness, and willingness to learn are essential factors in their development of leadership competence [1].

### Leadership development programs

In recent years, there has been an increased emphasis on evidence-based leadership and leadership development programs (LDPs), particularly in high-income countries [2]. The focus of leadership development has shifted from individual competence acquisition toward collective, organizational learning [1, 9]. This reflects the demands of healthcare as a complex system where improvement interventions must be context-sensitive and socially embedded [10, 11]. However, social and collaborative learning processes—essential for navigating complexity—are often underdeveloped in healthcare settings. Research has shown that leaders at times see each other more as competitors than colleagues, weakening shared learning processes [7, 11]. LPDs are criticized for lacking a theoretical foundation and effect [12, 13].

Despite growing investment in leadership development programs (LDPs), there remain persistent uncertainties regarding how these initiatives are pedagogically conceived, how their outcomes are evaluated, and how their effectiveness is understood and interpreted within organizational practice [14]. Several studies further illuminate how LDPs are often criticized for lacking a coherent theoretical foundation and for failing to demonstrate clear effectiveness, a critique that underscores the conceptual ambiguity that continues to characterize parts of the field [12, 13]. Although targeted competence development for nurse leaders has been shown to yield positive outcomes, the field still lacks nuanced insight into which pedagogical and relational approaches most meaningfully support leadership development in practice [4, 8, 15] essential for supporting effective leadership development [16].

### Theoretical perspectives on leadership development

Learning is understood as a purposeful and facilitated process through which individuals acquire competencies including knowledge, skills, attitudes, and behaviors. This learning process requires deliberate pedagogical and didactic planning [17]. Leadership development and competence-building depend on learning that is self-directed, experience-based, and embedded in leaders’ professional contexts [18, 19]. This pedagogical orientation is best described as andragogical, consistent with Knowles’ [19] assumptions that adult learners draw on prior experience, pursue problem-centered learning, and take an active role in directing their own development [19]. These andragogical principles are complemented by the descriptions of transformative learning theory. Mezirow [20] posits that adults revise established meaning perspectives through critical reflection and dialogue. This theory offers a robust framework for facilitating deeper reflection within adaptive systems and encompasses rational, extra-rational, and emancipatory dimensions [11, 21].

Transformative learning is stimulated when assumptions are challenged, often through disorienting situations. New understandings are to be collaboratively constructed in safe social learning environments. This process enhances leaders’ autonomy, adaptive thinking, and capacity to navigate complex organizational contexts [21]. In leadership development, transformative learning often unfolds through bottom-up and incremental processes at individual or team levels and requires engagement with healthcare as a nonlinear and emergent system [11]. While explicit learning arises through structured pedagogical approaches, much adult learning also occurs implicitly and unconsciously [17].

Learning is shaped by the interaction of individual agency, behavior, and environmental influences, and is informed by prior experiences, expectations, goals, and attitudes. Leaders’ development can be supported through performance feedback, behavioral modeling, and verbal encouragement from colleagues and supervisors [22]. Leadership learning is therefore both cognitive and social, unfolding incrementally through relational processes that integrate intrapersonal, interpersonal, and collective development [11].

Empirical evidence demonstrates that leadership development is strengthened by action-based learning, feedback, self-development, and pedagogical strategies such as small-group work, project-based learning, mentoring, and coaching [1]. In such contexts, safe-to-fail environments play a critical role by supporting critical reflection and innovation, enabling leaders to question established practices and explore new ways of working [11].

This perspective aligns with the theoretical model *The House Created with Caring in Leadership* [8] which positions caring as the core of nursing leadership. Caring for patients is understood as inseparable from caring for staff and oneself as a leader. The model frames leadership within organizational structures while emphasizing leaders’ need for support and development through reciprocal relationships in the leadership hierarchy. Six premises underpin caring leadership: awareness, movement, position, values, competence, and community.

Our philosophical and theoretical stance is rooted in a relational ontology, which holds that knowledge arises through affirmative, dialogical, and co-constructed processes [23]. From this viewpoint, appreciative inquiry contributes that understanding is fundamentally relational and evolves through interactions that highlight strengths and possibilities within relationships [24]. This approach emphasizes recognizing and enhancing values and capabilities in organizational settings, viewing reality as relational [25] and shaped by multiple, context-dependent interpretations [23, 24].

This study is the second study in a three-study action research project aimed at co-creating a knowledge-based, continuous LDP for healthcare leaders in a rural Arctic municipality. The project was initiated by the municipality as a response to a larger re-organization process.

Situated within a hermeneutic tradition, each study expands the understanding of the phenomenon and informs the following [26]. The project follows a bottom-up approach in which participating healthcare leaders act as co-creators; their experiences, interpretations, and practice-based insights shape the LDP across all three studies: The first study explored leaders’ existing knowledge and experiences to develop a common basis for co-creating the LDP program. Two themes were identified: (1) changing from striving solo players to team players, and (2) learning to handle a conflicting and complex context [7].

These findings informed the development of the LDP by highlighting the need for a collective and relational learning process rather than an individually oriented, competency-based approach. The findings also generated new understandings that shaped the focus of the present, second study, which explores the participants’ experiences with pedagogical and relational principles applied in workshops during LDP development. The forthcoming third study will present the ultimate results of participating in the LDP from the participating leaders’ perspectives and thereby completing the hermeneutic movement from initial understanding to a more integrate, comprehensive interpretation of the process of co-creating a knowledge-based, continuous LDP for healthcare leaders in a rural, Arctic municipality.

## Methods

### Study design and methodological rationale

**Fig. 1:**
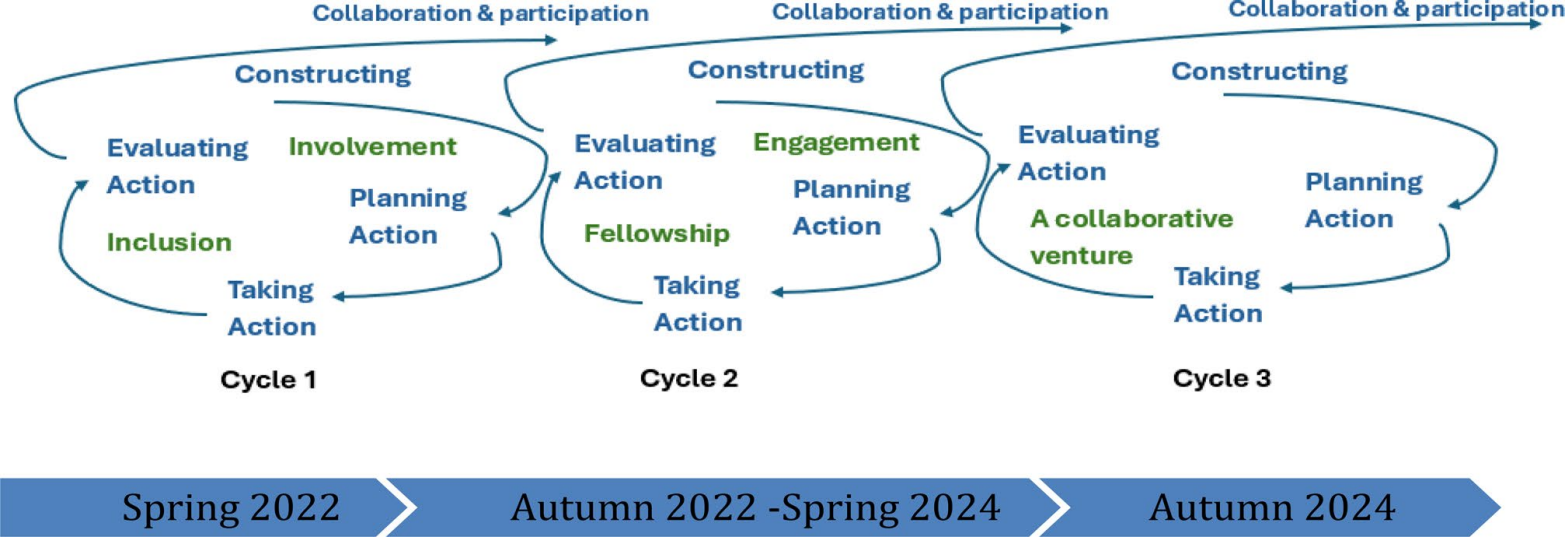
Spiral of action research cycles

Action research (AR) was chosen as the methodological framework in response to the municipality’s need for practice-embedded research that facilitates organizational change through collaboration and co-creation [27, 28]. Its participatory structure engages practitioners as co-researchers through iterative cycles of constructing, planning, acting, and evaluating (Fig. 1), fully aligning with this study’s co-creative design [29–32]. As shown in Fig. 1, these cycles involve collaboratively framing issues, jointly designing context-sensitive actions, co-enacting interventions, and appraising both intended and emergent outcomes to guide subsequent iterations. Throughout the process, critical reflection and appreciative recognition of participants’ experiences serve as the connective material between phases, sustaining learning, recalibrating assumptions, and ensuring that the process remains collaborative and cumulative [27].

In this cyclical process, participants were actively involved in shaping both the LDP and the research process, following a participant-based paradigm where knowledge is constructed through relationships and dialogue. Incorporating participants’ perspectives and experiences fostered mutual learning, shared understanding, and relational equality, while influencing which topics and knowledge were prioritized [33, 34]. The pedagogical and relational principles utilized were based on five core appreciative principles [35], and in addition a sixth principle: caring and psychological safety developed by Magnussen [36] as briefly described in Table 1.

**Table 1:** Core principles outlined by Cooperrider et al. [35] and Magnussen [36], along with a description of how each principle was applied in the study

Core principles	Short description	Principles applied to this study
1. Constructionist	Language and conversation, how words shape the world	Participants’ personal and professional experiences and successful situations in everyday language
2. Simultaneity	Asking a question denotes change	Engagement in appreciative conversations and reflective practice enhances self-awareness and cultivates greater sensitivity toward others.
3. Anticipatory	Hopeful image of the future determines a more positive present day	The articulation of shared vision activates reflective and creative thinking, thereby informing the design of interventions. Appreciative language has a generative and contagious quality, contributing to the co-construction of a positive discourse.
4. Positive	Generating momentum through positive questions	The use of positive language facilitates new ways of thinking, generates emotional uplift, and encourages participants to engage in mutual sharing.
5. Poetic	The choice of what to study affects what will be created	The sharing of successful and meaningful experiences facilitates critical reflection on individual practice and fosters the co-construction of new understandings and practices within the professional community.
6. Caring and psychological safety	Creating relationships and responsibility, and the desire for others to succeed	Mutual recognition and care contribute to the development of safety, trust, courage, personal growth, and a sense of responsibility—factors that enhance understanding of workplace dynamics and the value of collaborative relationships.

Following this design, the researchers and participants have worked closely together. The researchers have facilitated actions based on methodological principles and generated knowledge about these actions trying to bridge the gap between theory and practice [35].

### The workshops’ context and structure

This AR project formed part of a broader organizational reform in a rural municipality in Norway. Senior leadership engaged the authors to design a research-informed intervention aimed at enhancing leadership competencies in healthcare. The LDP was supported by the municipal department of human resources. The municipality has approximately 11,600 residents [37] and provides healthcare services through nine units and sixteen departments, employing around 500 staff.

Based on previously described pedagogical and relational foundations for this study, the LDP was conducted as two-day workshops held twice annually; one in the spring and one in the autumn in total six workshops in this two-year period from 2022 to 2024 (Fig. 1). Each workshop took place at conference venues outside participants’ workplaces to foster personal and professional interaction and networking [8]. The venues included a larger room for gathering the whole group, group rooms for collaborative work and focus group interviews (FGI), and nearby cabins that accommodated 2–4 participants. Further, the development of social relationships and a sense of psychological safety was emphasized through group composition, the selection of practical exercises, and evening social events with a quiz. Shared meals, breakfast, lunch, and dinner were arranged.

The agenda for the workshops included a mix of short lectures incorporating participants’ experiences and theoretical presentations, practical exercises, and guided group reflection. Ample time was allocated for breaks to encourage dialogue and relationship-building [7]. The workshops followed a recurring structure, with small adjustments in accordance with feedback from the participants, detailed in Table 2.

**Table 2:** Example of a workshop structure

Program Element	Associated Practices
Breakfast	Informal conversations among participants
Feedback Session	Review and discussion of previous survey results
**Theme Introduction**: Development and Collaboration **Theory lecture**: Team and Team Management (30 min)	Facilitator-led lecture with PowerPoint; participants ask clarifying questions
**Group Exercise** **(3–5 participants).** **Experience Sharing**: Successful or Unsuccessful Collaboration	Participants share impactful experiences (narratives); peers listen without interrupting; group discussion based on participant questions; plenary reflection
Break and Mingling	Informal conversations
Lunch	Shared meals with open seating to encourage interaction
**Theory Session**: Leadership and Collaboration (20 min)	Oral presentation supported by e.g. PowerPoint
**Reflection in Triads**: Collaboration Between Units	Facilitator assigns task; roles clarified; reflections shared in small groups and plenary
**Group Exercise**: Team Compass, tower	Case-based role play; facilitated using “Leading with Questions”; iterative questioning to support problem-solving; group discussion
Dinner	Informal conversations
Quiz Activity	Informal engagement and relationship-building through playful interaction

The authors entered this study with diverse pre-understandings, shaped by extensive professional experience in healthcare services within rural Arctic municipalities, including in leadership roles. Two of the authors TAH and LS had previously led an LDP in the municipality under study [38, 39], and TAH are currently employed there. RS participated in the earlier iteration of an LDP, while ILM has led other AR initiatives in similar rural healthcare contexts. Collectively, the authors acknowledge the complexity and variability of leadership practices in municipal healthcare, often characterized by fragmented structures and insufficient follow-up mechanisms. Drawing on prior research and practical experience, particularly the doctoral work of TAH and RS on healthcare leadership development [8, 40], the study is informed by a nuanced understanding of the challenges and opportunities inherent in leadership development in rural healthcare settings. In addition to the author-facilitators (RS, TAH, LS and ILM), one external facilitator, EB, an HR- advisor in the municipality, participated in the workshops. He was identified through the participating organization and selected based on his experience with group facilitation and his familiarity with the topic and context. All facilitators were chosen for their competence in managing group processes and ensuring balanced participation. The author-facilitators also have extensive experience both as leaders, with developing and leading quality improvement projects in municipal healthcare services, and as researchers within this field.

### Participants

All leaders across three hierarchical levels within healthcare services in the rural Arctic municipality were invited to participate in the study, including leaders appointed during the project period. Participation in the LDP was voluntary and open to all leaders, regardless of their involvement in the AR project. All invited leaders initially accepted the invitation, although some were unable to attend specific workshops due to illness or vacation. Further details regarding participant characteristics can be found in Table 3.

**Table 3:** Participant characteristics (*n* = 41)

Number	Profession	Further education	Leadership level	Age	Gender	Leadership experience
27	Registered nurse (RN)	Master’s degrees: 7 Postgraduate education in leadership: 11 Clinical postgraduate education: 8 Pedagogy/supervision: 2 Therapy/Communications: 2 ICT: 1	15 first-line leaders 10 middle leaders 2 senior leaders	36–63 years (mean = 46)	25 women 2 men	1–28 years (mean = 10.2)
5	Social educator	2 health leadership 1 coaching 1 pedagogical guidance	4 first-line leaders 1 middle leader	37–59 (mean = 45,8)	4 women 1 man	3–19 years (mean = 9.6)
2	Physio- therapist	1 master in physiotherapy science	2 first-line leader	32–37 (mean = 34,5)	1 woman 1 man	4–6 years (mean = 5)
1	RN and lawyer	none	1 senior leader	46	1 woman	6 years
1	Child protection educator	pedagogical guidance, substance abuse care	1 middle leader	57	1 woman	28 years
1	Preschool teacher	health, environment, and security	1 first-line leader	57	1 woman	23 years
1	Social worker	public policy and administration health leadership	1 first-line leader	51	1 man	15 years
1	Nurse assistant	none	1 first-line leader	62	1 woman	8 years
1	Physician	personnel leadership and competence development	1 middle leader	34	1 woman	2 years
1	Treatment pedagogue	health leadership, substance abuse care and psychiatrics, psychology	1 first-line leader	42	1 woman	15 years
Total:						
41 participants	10 different professions	30 different further educations	3 leadership levels	32–63 (mean = 47,6)	36 women 5 men	1–28 years (mean = 10.5)

### Data gathering

In accordance with AR methodology [27], multiple qualitative data collection methods were used:

#### Semi-structured focus groups

A total of 16 FGIs were conducted in private rooms on the second day of each workshop. Each session lasted approximately one hour and was audio recorded. Participants self-organized into groups ranging from 5 to 13 individuals. TAH, RS, ILM and LS alternated on moderating and co-moderating the sessions using semi-structured thematic interview guides with open-ended questions [31]. These were developed collaboratively by all authors, informed by prior research [7, 8], and based on principles of AR [41] (Appendix 1).

#### Participatory observations

Participant observation was conducted during group work, scenario training, plenary discussions, and FGIs. RS and ILM documented these observations through unstructured field notes aimed at capturing communicative, relational, and interactional dynamics. The notes followed no predefined format. The primary purpose of the observations was to explore how leaders planned and facilitated group work, with attention to role distribution, communication, behavior, and group dynamics, and how they responded on the pedagogical and relational methods facilitated by TAH, LS and an advisor from the municipal department of human resources. The observations provided complementary data to the spoken word and enabled insight into a broader range of behaviors, including actions, informal interactions, and open discussions [42].

#### Online evaluation surveys

After each workshop, participants completed anonymous online surveys. These surveys provided feedback on workshop content and structure and informed the planning of subsequent sessions.

### Data analysis

The overall data were analyzed inductively using Braun and Clarke’s [43, 44] reflexive thematic analysis (RTA), to identify and interpret patterns of meaning within the dataset in relation to the study’s aim. Here, ’inductively’ refers to an approach in which the analysis is grounded in the data, rather than representing a form of pure induction [44]. By constructing themes, our aim is not only to describe the data but to offer insights into patterns in the participants experiences related to the relational and pedagogical methods used in the LDP. The researchers’ role in knowledge production is central in RTA and themes are understood as ‘creative and interpretive stories about the data, produced at the intersection of the researchers’ theoretical assumptions, their analytic resources and skills, and the data themselves [45, p. 594]. RS, ILM and TAH manually transcribed the recorded interviews. Handwritten field notes were digitized by RS and ILM and integrated into the dataset, alongside the survey responses. The dataset was manually organized in Microsoft Word and analyzed in Norwegian; final themes and illustrative quotes were subsequently translated into English.

**Fig. 2:**
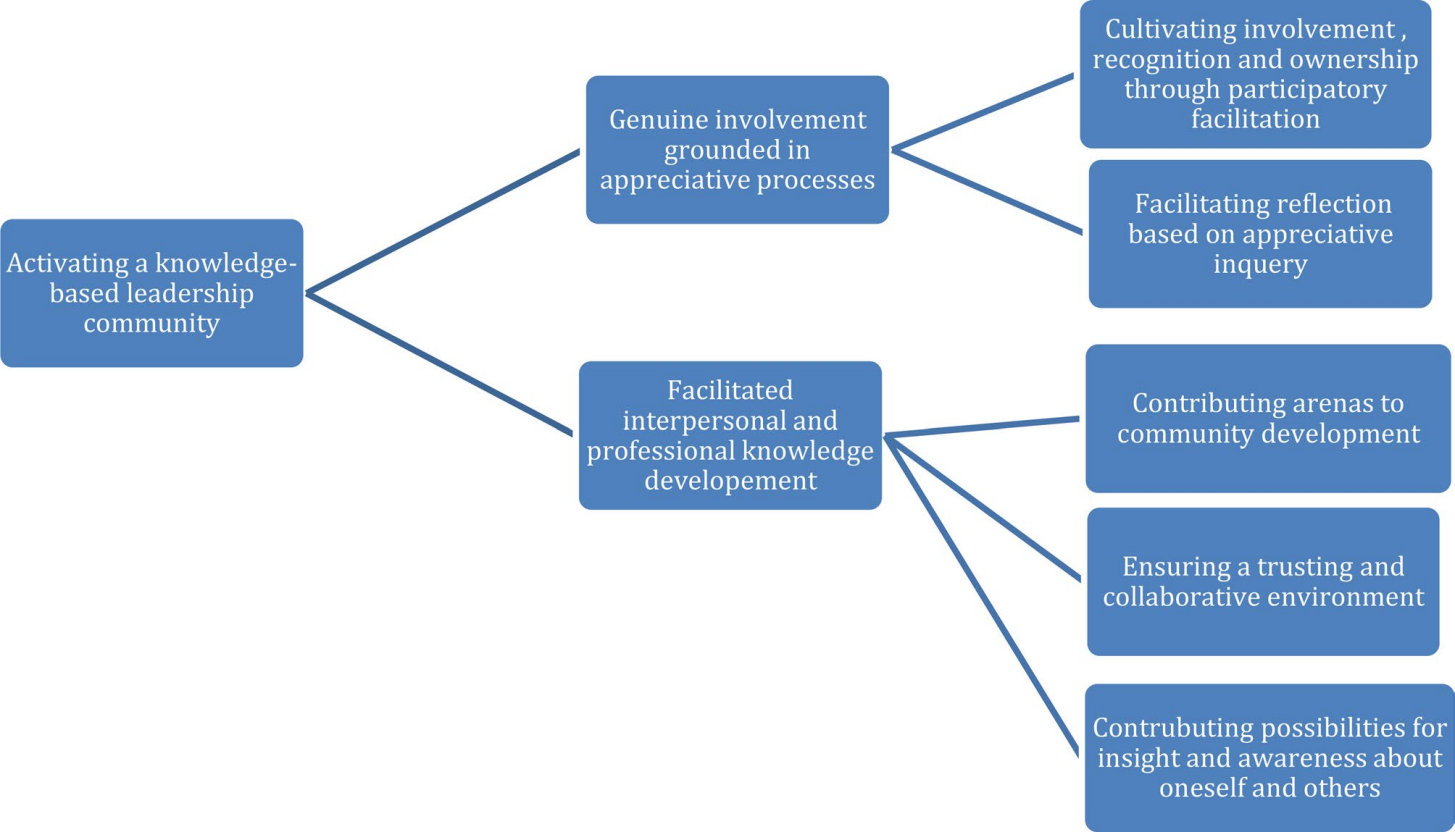
The overarching theme, themes and subthemes (phase five [43, 44])

ILM led the analysis in close collaboration with the other authors following Braun and Clarke’s [43, 44], six steps:

1. Each author independently familiarized themselves with the transcripts through reading and re-reading, noting statements, situational descriptions, and short narratives relevant to the research questions. For instance, comments on relationship-building and communication were preliminary grouped.
2. All notes were systematically labeled, and initial codes were generated with attention to both semantic and latent meanings.
3. Codes were clustered into potential sub-themes by examining relationships and patterns among them, representing broader patterns across the dataset.
4. Themes were carefully reviewed and developed through abstraction. This included revisiting transcripts and notes to ensure completeness and coherence, evaluated using Braun and Clarke’s 15-point checklist [43].
5. Each theme was clearly described and named reflecting their core meaning and significance.
6. The final themes formed the foundation of the results chapter, supported by selected participant quotes (Fig. 2).

The interpretive process was non-linear, involving frequent movement between phases and regular reflexive meetings among the authors to deepen understanding of leaders’ experiences and achieve a shared horizon of meaning [26]. Preliminary findings were presented to participants during workshops as part of a member-checking strategy [46]. Feedback from participants was thoughtfully incorporated to enrich and deepen the emerging themes.

## Results

The study included 41 healthcare leaders: 3 senior leaders, 13 middle managers, and 25 first-line managers. The participant group represented a high level of professional expertise. Collectively, they held 10 different bachelor’s degrees and 30 post-graduate qualifications, 17 of which were related to leadership. Seven participants held master’s degrees. Leadership experience was also substantial, with an average of 10.5 years across the group. The gender distribution included 36 women and 5 men. This diverse and experienced group provided rich insights into leadership practices in rural municipal healthcare, contributing to the depth and relevance of the qualitative analysis. An overview of participant characteristics is presented in Table 3.

The analysis identified an overarching theme: Activating a knowledge-based leadership community, and two themes: (1) Genuine involvement grounded in appreciative processes (2) Facilitated interpersonal and professional knowledge development (Fig. 2).

### Genuine involvement grounded in appreciative processes

The leaders expressed a strong sense of appreciation stemming from how they experienced being genuinely involved in developing the workshop programs. They felt heard and included in the planning process when offering suggestions and feedback—an experience they contrasted with previous LDPs they had participated in, where the content had been predetermined. This active engagement made the sessions feel both familiar and predictable, combining theoretical lectures with practical exercises that were experienced tailored to their needs. They further described the facilitation approach as both appreciative and hierarchy-flattening, emphasizing that, regardless of participants’ educational backgrounds or leadership positions, it fostered a sense of inclusion, motivation, and sustained engagement in their ongoing leadership development. Many participants noted feeling privileged to co-create the development of the LDP. This theme is supported by two subthemes: (1) Cultivating involvement, recognition and ownership through participatory facilitation and (2) Facilitating reflection based on appreciative inquery.

#### Cultivating involvement, recognition and ownership through participatory facilitation

Participants consistently described the LDP as grounded in co-creation, enhancing their involvement, sense of mutual recognition, and ownership. The participant-led content design contributed to a facilitation approach experienced as responsive and relational. Frequent comments such as *‘We are heard and taken seriously’* illustrated how this approach fostered relevance, motivation, and pedagogical adaptability. The opportunity to influence the structure of the program was highlighted as both affirming and empowering. As participant 13 elaborated:

I think it’s so great that we, as leaders and participants, are involved in setting the agenda for the meetings. I really appreciate being able to suggest what we need and the program is very important to me….makes you feel valued (FGI 1)

An appreciative facilitation approach was described as enjoyable and meaningful, enhancing engagement, self-awareness, and the ability to articulate professional experiences, as described in the FG. While several participants were initially hesitant to speak in groups, many gradually embraced the opportunity; even those reluctant reported challenging themselves when encouraged. Structured time for reflection, discussion, and exchangefollowing practical exercises supported this development.

The facilitators contributed their own perspectives while inviting participants to share theirs, creating a climate of trust and active commitment. Participants consistently described the workshops as safe and supportive spaces where honest feedback and shared experiences promoted psychological safety. This interaction fostered a sense of mutuality in which participants learned from each other and viewed facilitators as equal conversation partners with relevant leadership experience. This dynamic was observed as egalitarian, extending beyond formal leadership hierarchies and reinforcing a shared learning environment.

Reflection tasks closely aligned with everyday leadership practice enabled participants to see their own experiences acknowledged and understood. Through this, they reported increased awareness of their leadership roles, recognition of behavioral patterns, and clearer areas for improvement. A commonly emphasized trajectory was the shift from initial hesitation to growing confidence and insight—experienced as a meaningful part of their continuous leadership development.

#### Facilitating reflection based on appreciative inquiry

Participants described the workshops as a welcome pause from daily pressures. Being physically distanced from their everyday workplaces provided space for sharing knowledge and experiences, enabling deeper conversations and renewed perspective. The pedagogical structure—short theoretical inputs, personal presentations, and structured group reflection—was consistently highlighted as impactful. Group assignments introduced practical communication tools and conversation aids that participants found directly applicable to their leadership practice. They emphasized the value of sharing both successes and challenges. Observations showed that participants’ presentations held the group’s attention and were followed by affirming comments. As participant 6 reflected:

It is very powerful to hear others’ experiences—good and bad. I get an expanded understanding of the person and the situation. I am touched and do experience this as an oasis. The methodology encourages reflection. I like the format. (FGI 2)

A shared theoretical framework provided a common language for interpreting leadership experiences and grounding reflective dialogue. Participants described the exchange of practices and perspectives as both educational and affirming, often emphasizing how much they learned from one another. Exercises stimulated reflection, both in the moment and afterward. As one example, one exercise required guiding a blindfolded partner, illuminating emotional and relational dimensions of leadership. Participants reflected on how clear and close guidance fostered safety, while distant or vague leadership led to discomfort and confusion. As one participant stated during the exercise:

Leadership is about meaning. You have to understand for yourself what you are going to see. (Fieldnotes, workshop 2)

Participants consistently highlighted the facilitators’ relational methods, especially their encouragement of open sharing, as crucial for creating a psychologically safe environment. Plenary discussions revealed diverse reactions, from laughter to critical reflection, underscoring both the artificial nature of exercises and their resonance with real leadership dilemmas. As participant 3 remarked:

Good to have something to sharpen your skills on—dare to disagree in order to develop and learn. Good questions give new ideas. (FGI 5)

This safe atmosphere enabled participants to speak vulnerably about professional and personal challenges, including conflict management, staff turnover and difficult conversations. Several emphasized the importance of being ‘on the same page’. Sharing experiences increased their confidence and strengthened their capacity to engage in future conversations with employees and colleagues. Articulating leadership practices helped them recognize and validate their own competencies. As participant 5 summarized:

This is where I learn my lessons. I have had a lot of personal growth…. (FGI 3)

Embodied exercises, including roleplay, revealed the emotional and relational dimensions of leadership. Survey responses indicated that while some found roleplay uncomfortable, many acknowledged its value by learning from being challenged outside their comfort zone. Participant 9 in FG5 said:

Presenting was difficult for me to begin with. When I also have to talk about something I have done but not fully succeeded with, it becomes uncomfortable. It is hard to find the right words when everyone is looking at me. That is why it feels overwhelming when I receive unanimous support from the group afterwards. I grow from that!

Roleplay was introduced early in the LDP, and retrospective reflections suggested it might have been more effective after group cohesion developed. Participants emphasized that interpersonal familiarity and psychological safety were critical for engaging meaningfully in role play. While some exercises felt constructed, they were nonetheless evaluated as meaningful and transferable to real-world practice. Participant 37 remarked:

This was smart! I hadn’t thought of that before. (FGI 6)

The alternating rhythm of small-group sharing and plenary dialogue was described as affirming and thought-provoking. Observations showed high engagement, with group tasks and facilitation fostering laughter, energy, and a positive atmosphere. Spontaneous discussions often replaced breaks. For newly appointed leaders, the LPD also functioned as onboarding. One participant appreciated colleagues being “consistently helpful and eager to share,” and another followed up:

It is a way of welcoming people into the warmth, a profound enrichment—meeting colleagues and reflecting on leadership together. (FGI 10/P32)

Across the group, the LPD was regarded as a positive arena for professional reflection and peer support—a ‘good place to be’. Participants underscored the importance of mutual reliance, noting that ‘we need each other’. The program fostered joy, renewal, and strength, described metaphorically as ‘armor’ in navigating leadership challenges and organizational change. The emphasis on methods highlighting relational connectedness and shared reflection appeared to be central to participants’ positive experiences and their perceived readiness to engage with the demands of their leadership roles.

### Facilitated interpersonal and professional knowledge development

Participants described how shared meals, social activities such as the evening quiz, and the shared living arrangements made it easier to connect across units and leadership levels, both formally and informally. Survey responses reinforced this, highlighting that the overall atmosphere enabled open and trusting communication. Further, participants reported feeling safe to explore and share, noting that the facilitators modelled trust-based communication through interactions characterized by a light and playful tone combined with deep mutual respect. Expressions such as ‘getting a little push’ and ‘wishing each other well’ reflected the friendly and generous dynamic that developed within the group. While the academic components were highly valued, participants emphasized that the social dimensions were equally important for building relationships, fostering belonging, and strengthening professional ties. Together, these experiences supported both interpersonal connection and professional knowledge development. This theme is substantiated by three subthemes: (1) Contributing arenas to community development (2) Ensuring a trusting and collaborative environment and (3) Contributing possibilities for insight and awareness about oneself and others.

#### Contributing arenas to community development

Participants consistently emphasized that dedicated arenas played a central role in developing a sense of community throughout the leadership development program. Both formal and informal settings contributed to progression from initial interpersonal distance to increasing trust, cohesion, and belonging.

Participants highlighted those structured activities such as theory sessions, group assignments, and guided reflections offered predictable, purposeful spaces for interaction. Working closely with both familiar and unfamiliar colleagues helped break down interpersonal barriers, fostering deeper professional and interpersonal relationships. Although some found the reflective elements challenging, many valued the opportunity to present different sides of themselves. As participant 22 in FGI 9 noted:

I’ve drawn energy from this arena and from being together in this dynamic….

Participants described the FGIs as valuable arenas for reflecting on their own strengths. While the reflective process was sometimes demanding, many articulated competencies such as delegation, promoting well-being, cultivating informality, caring for others, identifying potential, maintaining continuity, and embracing vulnerability. Participant 2 described this increased self-awareness:

I am a present leader because I know the organization. I am a real support for the employees…. everyone is lifted up in some way…. I am now aware of that. (FGI 6)

Informal settings were described as just as crucial for cultivating a sense of belonging. Overnight stays, especially those in cabins with shared rest and living rooms rather than hotels—were perceived as particularly conducive to closeness and continuous conversation. When such shared settings were absent, the experience often felt fragmented, interrupting relational processes that were otherwise developing. As participant 32 noted:

….we rarely have spaces where we can meet and talk…….

The evening quiz emerged as a surprisingly powerful social arena for building teams based on diverse strengths beyond traditional leadership roles. Led by one facilitator (LS), with others joining as team members, the quiz fostered collaboration, inclusiveness, and equality. Participants appreciated interacting with colleagues ‘just as themselves’, outside of formal roles. The goal was not simply to win, but to create a ‘winning team’ by recognizing and combining diverse abilities. Competitive instincts surfaced, yet participants emphasized the importance of team composition and the value of different strengths. Regardless of the outcome, the quiz generated laughter and positive energy that carried into the next day and became a recurring topic of conversation, reinforcing shared experience and strengthening bonds. Several participants reflected that the activity encouraged new ways of thinking about collaboration, diversity, and mutual support.

Over time, interaction patterns also changed. Participants initially preferred to sit with familiar colleagues but gradually became more deliberate in seeking contact across boundaries. Some intentionally chose new seating arrangements to broaden their relational networks. In later survey responses, participants expressed a desire for even more structured mixing, suggesting mechanisms such as pre-assigned quiz teams or place cards to encourage broader interaction. They viewed this as a way to strengthen informal networks among managers, making it easier to initiate contact, share knowledge, and seek support across organizational divides. As participant 5 in FGI 7 observed:

It’s good that we mix a bit. I think it’s a strength. You think new thoughts and don’t stick to the usual….

#### Ensuring a trusting and collaborative environment

The facilitators established an early foundation of safety, respect, and collaboration by introducing group tasks and social activities that modeled an appreciative approach. They encouraged openness by valuing diverse perspectives, acknowledging participants’ contributions, and framing tasks as shared endeavors. This invited active participation and mutual engagement, creating a psychologically safe environment where participants experienced a sense of community that allowed them to “be who you are,” even in a leadership role. This was associated with increased authenticity, greater openness, and the freedom to lead in ways aligned with personal values. Several participants described how the trust that developed during the LDP strengthened their confidence in their roles and in interactions with others—confidence they later applied when supporting leaders, colleagues, and staff. As participant 3 from FGI 2 expressed:

If you are secure in your role, and secure in what you are going to convey, then perhaps it creates security in others…….

Participants also reported that the pedagogical and relational approaches used in the program encouraged personal development, including improving public speaking, strategic thinking, and learning from mistakes. At the same time, the theme of “failure” was rarely addressed openly. To challenge this pattern, the facilitators introduced an explicit focus on acknowledging and learning from mistakes in Workshop 4. This was experienced as constructive and liberating, helping to normalize vulnerability as a natural aspect of leadership. Many participants noted that they found it easier to identify strengths in others than in themselves and described feeling joy and motivation when colleagues succeeded. This mutual recognition cultivated a supportive climate and reinforced the sense of collective growth.

The appreciative and reflective questions posed by the facilitators during the FGIs were experienced as affirming and constructive. Several participants emphasized that these conversations made their own strengths more visible—not only to themselves, but also to their colleagues. This increased visibility contributed to enhanced confidence and a stronger sense of ownership in their leadership roles. Being invited to reflect within a supportive group fostered authenticity and heightened awareness of their leadership practice, and the process strengthened their belief in their own leadership capacity—both personally and professionally.

#### Contributing possibilities for insight and awareness about oneself and others

Participants recurrently emphasized that the workshop structure played an important role in their learning and professional development. The theoretical sessions introduced new ways of thinking that were seen as meaningful, rewarding, and practically useful. Across leadership levels, participants described everyday communication in leadership as challenging, and they saw it as an area in need of improvement within their municipality. This made topics such as “the difficult conversation” and conflict management especially relevant. Collaborative group tasks with open questions encouraged reflection and increased participants’ awareness of their own communication styles and personalities. Several reported that this led to concrete changes in how they approached conversations with employees. They explained that the group work helped them recognize their personal strengths and limitations, reinforcing their understanding of the need to invest time in developing communication skills.

We become better leaders and communicators—and it helps us further. (FGI6, P24)

The theoretical components were frequently described as valuable input—conceptual anchors that helped participants make sense of their own practices and experiences. Many found the frameworks intellectually stimulating and relevant, nevertheless some also experienced unfamiliar terminology and abstract concepts as distancing. As one participant noted:

The way theory is presented and the language used can feel like a technique of domination. It needs to be brought down to a level where others can contribute…the concepts and words must be meaningful, not just a word but something everyone can understand. (Fieldnotes, workshop 3)

Clear, everyday language from the facilitators was therefore appreciated, as it fostered mutual understanding and shared meaning. Although some found the longer theoretical segments demanding, the value of theory was widely acknowledged for offering new perspectives on practice. As participant 4 put it:

I understand theory more deeply when it’s combined with activity. I can reflect, try out, criticize, and dare to challenge myself. (FGI 4)

Researcher observations indicated that when participants’ leadership competencies and tacit knowledge were integrated with theoretical input, their engagement increased, and their understanding of leadership concepts deepened. Through reflective dialogue, tacit knowledge became explicit and shareable, contributing to collective learning and leadership development. The interactive pedagogical approach was experienced as fostering personal growth, enabling participants to gain new professional insights and uncover aspects of themselves they had not previously recognized. These insights were carried into their workplaces, where participants felt better equipped to experiment with and apply the methods introduced.

## Discussion

This study explored healthcare leaders’ experiences with pedagogical and relational principles, used in workshops to develop an LDP in a rural Arctic municipality. The overarching theme- Activating a knowledge-based leadership community”, was underpinned by two main themes: *Genuine involvement grounded in appreciative processes* and *Facilitated interpersonal and professional knowledge development.* In this following discussion, we will deliberate how these findings contribute to existing knowledge, clarified within three elements: (1) Creating space to develop (2) Inviting to reciprocal knowledge exchange and (3) Contributing to a changing culture. A hermeneutic process allowed us to develop these elements, merging insights from existing research, previous studies [7, 8], and the findings of this study, pedagogical theories [11, 17, 24], and leadership theories [2, 4, 5, 47].

### Creating space to develop

Prior research shows that locating innovation hubs outside regular workplaces can contribute new ideas and creativity [11]. This study extends that understanding by demonstrating how the physical and social design of workshops fostered connection. Arranging LPD workshops in neutral, off-site venues enabled undisturbed engagement in formal and informal activities and promoted interaction across organizational levels. This can be understood as the deliberate creation of a form of social architecture—not merely a contextual frame, but a purposeful mechanism used to cultivate relational dynamics and support collaborative learning. This dimension is found to be under described in the LPD literature [18] yet our findings indicate that the social architecture is equally as significant as the academic and content-focused components of the program. These findings align with Faller and Marsick’s [11] view of communities of practice, where shared spaces and joint participation deepen relationships and learning.

The significance of physical space in leadership development remains underexplored. Although immersive settings are associated with enhanced engagement, evaluations often overlook contextual factors such as location [14]. Burton et al. [10] highlights healthcare as a complex service where environmental and logistical conditions can unpredictably affect participation. In our study, inability to stay overnight (or proximity to home) disrupted interaction and continuity. These findings suggest spatial arrangements are not merely logistical but shape relational and pedagogical dynamics. While pedagogical principles remained the same, changes in relational dynamics interfered with the pedagogical approach, showing that spatial design influences both relational and pedagogical contexts.

The chosen physical setting facilitated the relational dimension of the workshops through shared meals, informal activities, and structured reflection, fostering trust and psychological safety. These seemingly minor activities supported relational leadership and collective meaning-making, aligning with Chen et al. [48], who stresses the value of informal learning arenas in leadership training. Principles of appreciation, involvement, and psychological safety permeated both formal and informal contexts, enabling lasting relational bonds.

Notably, the informal evening quiz became a success. Initially, it was intended as entertainment thus it proved to be far more significant for the pedagogical and relational approach than originally anticipated. The participants became acquainted with one another in an informal, trust-building manner, and reported that they developed an ability to select group members based on individuals’ unique competencies, irrespective of prior acquaintanceships or existing leadership structures. This practice extended beyond the program, enhancing collaboration in everyday work.

Nevertheless, Cummings et al. [4] caution that leadership development depends not solely on setting but also on facilitation quality and content relevance. While our study underscores the value of external venues, such choices incur higher costs compared to municipal facilities without accommodation or catering. This raises concerns about feasibility across municipalities with differing resources. Moreover, our findings highlight that the challenge may vary locally and poses particular challenges in rural and decentralized contexts [49].

### Invitation to reciprocal knowledge exchange

The LDP’s varied group tasks created spaces for participants to share personal insights, which were valued as meaningful contributions to collective learning. What emerges from this study is that participants developed a form of collective capacity and knowledge practice, shifting from building knowledge solely as individual leaders to establishing a shared knowledge base [7]. The study further highlights how participants described this collective capacity as a form of *‘armour’*—a protective resource that equips them to manage everyday pressures and navigate organizational change. This strengthening effect underscores the role of the LDP as an onboarding mechanism for newly hired staff, offering them not only conceptual and procedural knowledge but also access to a relational and collective foundation that supported their integration into the organization. The reciprocal exchange fostered connection and two-way communication, supporting individual reflection and group development. These findings reinforce research highlighting the role of dialogical arenas in leadership development, where vulnerability and openness are met with recognition [1, 3]. However, sustaining such arenas depends on organizational culture and structure, which may not consistently support openness and psychological safety [50].

This study shows how facilitated social arenas, such as shared meals and informal activities, helped foster connections that evolved into professional dialogue and reflection. Participants actively contributed through experience-sharing, presentations, and involvement in program planning. Although some found it challenging to present their experiences before a broader audience, the participants described this challenge as being positive, as it was found to be supportive and developmental. These findings support existing research that has described the importance of psychological safety and supportive environments in leadership development [1, 4]. Some studies have cautioned that such environments must be intentionally cultivated, as hierarchical structures and time constraints may hinder open participation [14, 48]. However, our findings show that intentional facilitation can promote organizational learning, beyond top-down or predefined approaches.

Group work and reflection in mixed groups were in our study considered particularly educational. Participants became acquainted with colleagues they had previously known little about, and the experience was described as developmental for both individuals and the group. These relational processes were observed and commented on in both professional and social settings, including informal activities such as quizzes. The reciprocal knowledge-exchange was also facilitated by the quizzes, that as previously described introduced a new dynamic into the group of participants. By disrupting established organizational structures, participants formed new teams across hierarchical and departmental boundaries to solve tasks together. This fostered unfamiliar collaborations and helped build new relationships. Such social learning environments contributed to leadership development as described in social constructivist theory, which emphasizes the co-construction of knowledge through interaction [22]. New insight is contributed by this study when concretizing what social learning environments can entail in practice. We show how informal, play-based elements like quizzes can foster trust, break down silos, and support cross-boundary learning areas that are found to be often overlooked in leadership development [38].

Principles from appreciative inquiry promoted active participation and reflection in this study, supporting transformative learning through recognition, relational engagement, and a strength-based approach [16, 21, 35]. This pedagogical approach appears to have activated participants’ intrinsic motivation and created conditions for growth. However, as noted by Faller & Marsick [11], transformative learning requires ongoing critical reflection and organizational support to be sustained over time. Our findings demonstrate that the choice of pedagogical and relational principles led to participants that experienced professional, personal, and relational growth, and expressed a desire to continue using focus groups as forums for reflection and discussion. This may indicate that relationships and networks must not only be activated but continuously maintained and monitored [8], ideally embedded in the organization’s strategic plans and annual cycles. Such integration is supported by studies emphasizing the importance of sustained collaborative structures in healthcare leadership [3, 7, 14, 38]. At the same time, this study reveals tensions between theoretical complexity and accessibility, and between structured pedagogical design and the need for flexibility and informality. These tensions are not seen as limitations, but as productive spaces for reflection and adaptation, core principles in action research [27] and hermeneutic methodology [26].

It is worth noting that some participants expressed uncertainty about whether the program would be sustained beyond the research period. A change in top-level leadership appeared to weaken the program’s organizational anchoring, raising concerns about long-term commitment and continuity. This highlights how leadership transitions can impact the institutionalization of development initiatives and underscores the importance of securing broad and lasting organizational support. If the LDP ends after this research period, the stream of input and energy to the leadership group also ceases. Leadership development is not self-sustaining; new leaders must be included and supported to become part of the team [2, 4, 8]. This study suggests that the LPD not only supported leadership development but also played a critical role in fostering a sense of belonging and psychological safety, particularly for those new to the organization. Discontinuing support may be demotivating and negatively impact service quality. Gathering and sharing experiences and capacities can motivate and support colleagues, which is essential for building leadership networks for personal and professional support [8]. This is echoed in studies that emphasize the role of peer learning and recognition in sustaining leadership motivation [1, 22].

Our findings indicate that FGIs served not only as data collection tools but as dynamic learning arenas where leadership, collaboration, and ethical awareness were actively practiced. Participants engaged with one another not just as professionals, but as people demonstrating openness, trust, and mutual recognition. These interactions reflected the pedagogical and relational principles of the LDP and were described as having professional, practical, and ethical value [5]. While previous research has emphasized the importance of ethical leadership [3, 49, 51], our findings suggest that ethics is not an additional competence but a foundational condition for human interaction and leadership development [15, 26]. Ethics emerged as a lived practice, cultivated through structured, dialogical spaces that foster trust and shared responsibility and embedded in the *process* of leadership development itself. This insight invites practitioners to rethink leadership development as not only a cognitive or strategic endeavor, but as a deeply relational and ethical practice one that requires intentional design to support trust, reflection, and shared responsibility.

The principles of participation supported interaction across all settings, whether in FGIs, group exercises, quizzes, or shared meals. No participant was left out; all were integrated into the LPD, both personally, socially and professionally in line with Solbakken’s recommendations [8]. This inclusive approach fostered a sense of belonging and collective identity among participants, which resonates with previous research showing a shift from leaders experiencing to be solo players to becoming team builders [7]. The approach also reflects broader organizational processes where leadership development moves from the individual to the collective, emphasizing shared responsibility and distributed leadership [14, 50].

### Contributing to a changing culture

A central mechanism in this development is the participants’ co-creation of the LDP. Instead of following a predetermined curriculum, the program was shaped through bottom-up processes in which participants collectively defined the thematic and professional content most relevant to their practice. This participatory design diminished formal hierarchical distinctions and generated an egalitarian interactional space where individuals across organizational and leadership levels contributed on equal terms. In practice, hierarchical boundaries were dissolved, enabling dialogue marked by reciprocal legitimacy, distributed agency, and shared ownership of the learning process. Such explicitly documented egalitarian conversational dynamics are rare in municipal LDPs; this absence reflects a broader gap in LDP research, which tends to emphasize competencies and program structure while providing limited empirical detail on the relational and interactional mechanisms through which learning unfolds [18].

As researchers, we do not understand this LDP as a final product or endpoint, but rather a foundation for continuity, sustainability, and the cultivation of a living practice. To sustain such practice, energy is required, and this energy was found by introducing AR. This understanding emerged from our results, supporting the principles from AR that describe how knowledge resides within each participant and can be activated when the right conditions are present [23, 35]. This study contributes with new knowledge, showing how our applied pedagogical and relational principles appear to have facilitated this activation, aligning with findings from transformative learning frameworks that emphasize participant-driven engagement [11, 32]. This study reveals tensions between theoretical complexity and accessibility, and between structured pedagogical design and the need for flexibility and informality. These tensions are not seen as limitations, but as productive spaces for reflection and adaptation, core principles in AR [27], and hermeneutic methodology [26].

Nevertheless, continuity could also depend on the participants themselves, who hold various leadership roles and serve as threads in the network. The network consisted of their professional and human competence, collectively forming a horizontal and vertical structure within the organization. Research highlights the need for collaboration both within and across units [5, 6]. As such, each individual participant carries collective competence, which when it is valued, may create conditions for growth through involvement, participation, and recognition [22].

Leaders need support and psychological safety to lead effectively [1] and are needed to develop leadership competence and reflect on their leadership style to manage complexity [4] and ethical challenges [3]. The applied pedagogical and relational principles in this LDP appear to have contributed to a movement toward knowledge-based practice, in line with transformative learning theory [16, 17]. This movement has initiated several interconnected processes, including cultural change, which is a well-documented outcome of action research in complex healthcare settings [40, 52]. The study further demonstrates that social and informal activities—as well as the arenas in which they occur—play a decisive role in shaping leadership practice. Relationships and competencies developed in these informal contexts are shown to transfer effectively into the formal arenas where leadership is enacted within the municipality, thereby reinforcing organizational learning across settings. Taken together, the workshops and the principles guiding them generate a distinct and recognizable cultural signature, characterized by strengthened relational dynamics, shared meaning-making, and an emerging collective orientation toward leadership practice.

### Strengths and limitations

A key strength of this study is its use of action research, which is inherently participatory. Participants were actively involved in the planning and implementation of the LDP workshops, facilitating a bottom-up development process and enhancing engagement [27, 50]. This approach also addresses common critiques of leadership development programs, which have been criticized for lacking a coherent theoretical foundation, by grounding the intervention in both practice and participant experience [12, 13]. Continuous adjustments based on participant feedback contributed to positioning participants as co-researchers, integrating research, development, and learning in ways aligned with the LDP’s pedagogical and relational principles [33, 34]. Including leaders from three organizational levels broadened perspectives and fostered mutual learning, although hierarchical differences and power dynamics may have shaped which topics were emphasized and how knowledge was prioritized [3, 34]. Participant turnover represented a limitation by affecting continuity and transparency over time. Ethical concerns related to autonomy and individualization required careful attention, though no serious issues were reported [32, 35].

Methodologically, the use of multiple data sources—focus group interviews, observations, and surveys—supported triangulation and enhanced rigor [32]. Although, focus groups ranged from 5 to 13 individuals, all participants were actively engaged and contributed to the discussions regardless of group size, and since they selected their own groups and were familiar with both the setting and each other, the facilitators did not observe any differences in the openness or depth of contributions across the focus groups. The extensive dataset carried a risk of overlooking nuances; however, participant involvement in data generation helped ensure that diverse perspectives were included [32, 35].

The facilitation team contributed expertise in pedagogy and leadership, with two facilitators and two researchers bringing research experience, and three researchers locally affiliated. This combination offered contextual insight while also requiring awareness of insider–outsider dynamics. Participant feedback on preliminary analyses further reinforced credibility by confirming that the interpretations resonated with participants’ experiences [42, 46].

We acknowledge that AR’s context-specific nature limits replicability. Nevertheless, detailed descriptions of organizational conditions, participant roles, workshop processes, and decision-making structures—together with visual figures—are intended to support transferability by enabling readers to determine relevance to their own settings, consistent with Lincoln and Guba’s [53] criteria for trustworthiness. Learning processes were shaped by contextual and relational factors, with participants making sense of and reshaping their environment through interpretation, adaptation, and emotional work. Appreciative Inquiry contributed by highlighting strengths and enabling generative, future-oriented dialogue [7, 16, 41, 43].

Reflexivity was an ongoing concern throughout the study. We recognized that our backgrounds, positionalities, and assumptions could influence interactions and interpretations. The team therefore engaged in continuous reflection to consider how our roles and organizational hierarchies might have shaped participation and analysis. While such influences cannot be fully removed, we sought to make them visible and remained attentive to less dominant contributions. Participant feedback on preliminary findings provided an important check on our interpretations and alignment with participants’ experiences.

## Conclusions and implications

Healthcare leaders in a rural municipality contributed valuable experience in co-creating a continuous LPD with emphasis on pedagogical and relational principles applied in the workshops. The insight indicated a unifying, developmental, creative, and learning-enhancing effect, activating knowledge-based practice through leadership development. The LDP was developed in close collaboration between participants and facilitators, not only inviting participation but actively realizing it. Participants recognized that their feedback was taken seriously and used to shape the LDP. This was an approach that was new to many and represents a unique contribution to this study.

This study demonstrates that knowledge-based leadership development is strengthened when multiple forms of knowledge—explicit, tacit, implicit, and declarative–procedural–conditional—are intentionally mobilized through structured reflection and collaborative learning. The findings further show that deliberate use of physical and social space, systematic facilitation of reciprocal knowledge exchange, and activation of the leadership community, including onboarding functions, contribute to building collective capacity. Additionally, organizational anchoring and continuity appear essential for sustainable cultural change, while integrating ethical and relational practice—centered on appreciative recognition—supports leadership development that is both context-sensitive and embedded in everyday practice.

The workshops were held at external conference venues, providing participants with the opportunity to step outside their everyday context and reflect on leadership from a broader perspective. A key strength of the study lies in its sustained focus on pedagogical and relational principles, applied consistently across both formal and informal settings over a two-year period. The findings suggest that these principles are well suited for the development of an LDP in rural municipal healthcare.

Importantly, the study positions the leader not only as a participant, but as a co-researcher, fellow human being, and professional—someone who carries new knowledge back into their own department and organization. This relational and participatory approach may have transferability to other municipalities and sectors beyond healthcare, both in terms of methodology and outcomes. Finally, the study provides a knowledge contribution to the further development of leadership programs and offers a foundation for future research and follow-up studies, particularly regarding long-term implementation and sustainability.

Future research should examine how the co-created LDP can be applied in other rural municipalities. Longitudinal and follow-up studies are also needed to assess its long-term outcome in leadership practice, teamwork, and organizational learning. Comparative work across municipalities could further clarify how local conditions and structures influence the implementation and outcomes of similar programs.

## Supplementary Information

Below is the link to the electronic supplementary material.

Supplementary Material 1

Supplementary Material 2

Supplementary Material 3

## Data Availability

The datasets generated and analyzed during the present study are not publicly available due to the confidentiality afforded by study participants but are available from the corresponding author upon reasonable request.

## Abbreviations

AR: Action Research
FGI: Focus Group Interview
LDP: Leadership Development Program
