# Supplementary material for: Activating knowledge-based practice through healthcare leadership development: insights from the second study of a broader action research project
Source: BMC Health Serv Res. 2026 Feb 25;26:444. doi: 10.1186/s12913-026-14243-5 (PMC13041005; doi:10.1186/s12913-026-14243-5)
Supplement: Supplementary file 1 — Supplementary Material 1 [file 12913_2026_14243_MOESM1_ESM.docx]

**Appendix 1, 1. Focus group discussion guide**

Aim: to explore how participating leaders experienced pedagogical and relational principles, used in workshops during the development of a continuous leadership program in rural municipal healthcare.

| **Background for the question** | **Research question** | **Question to the focus group discussion guide** |
| --- | --- | --- |
| Introductory questions | Participant characteristics | How old are you? |
|  |  | What is your basic professional education? |
|  |  | Do you have any postgraduate or advanced training? |
|  |  | How long have you been in a leadership position? |
| Opening questions | Experiences with the approach in the sessions and its consequences (bottom-up and appreciative) | What are your experiences with the way the sessions have been organized and conducted? |
|  |  | What are the consequences for you/us? |
|  |  | What difference does it make that we work in this way? |
|  |  | Does it make a difference? |
|  |  | Which seminar activities worked best? |
|  |  | How did they influence motivation and engagement? |
| Mapping questions | Experiences and reflections on the use of pedagogical and relationship-building activities and methods | To what extent does the use of quizzes contribute to leadership development among participants? |
|  |  | How do you perceive the role of dialogue during group work and informal breaks in fostering learning and collaboration? |
|  |  | How do you evaluate their own presentations, performance, and contributions within the learning context? |
|  | The significance of using theoretical lectures and individual presentations. | Advantages/ Disadvantages |
|  |  | Are we speaking the same language? |
|  |  | How do you think theory influences decision-making or leadership in your context? |
|  | Experiences and reflections on participation in action research, where you are both a participant and a co-researcher (within your own practice). | What are your thoughts on the bottom-up approach? |
|  |  | What are your thoughts on the appreciative approach? |
|  |  | Do you perceive yourself as part of the project and the leadership group? |
|  |  | Do you feel that the feedback you provide is incorporated into the program? |
|  | Experiences with inclusion. | When new participants join, how does that affect the group? |
|  |  | How do newcomers experience the process? |
|  |  | Previously, you described a shared value base for leadership development (trust, safety, respect): How do you think these values have been demonstrated or practiced during the sessions? |
|  |  | Do you have any wishes for the future regarding content or format? |
| Follow-up questions | How can we gain deeper knowledge within the different parts of the research project? | Can you provide more examples? |
|  |  | Can you elaborate further? |
|  |  | Do others see it the same way? |
|  |  | How did this happen? |
|  |  | How do you know this? |
|  |  | What led to good or poor outcomes, in your view? |
|  |  | Have you considered how this could be improved? |
|  |  | How does research-based knowledge contribute? |
|  |  | How does experiential knowledge contribute? |
|  |  | How does user knowledge contribute? |
|  |  | Does anyone have anything to add? |
