# Supplementary material for: Activating knowledge-based practice through healthcare leadership development: insights from the second study of a broader action research project
Source: BMC Health Serv Res. 2026 Feb 25;26:444. doi: 10.1186/s12913-026-14243-5 (PMC13041005; doi:10.1186/s12913-026-14243-5)
Supplement: Supplementary file 2 — Supplementary Material 2 [file 12913_2026_14243_MOESM2_ESM.docx]

**This study contributes to existing knowledge in several ways:**

It demonstrates how leadership development in rural and decentralized contexts can be strengthened through relational and participatory pedagogies.

It shows that psychological safety is not only a condition for learning, but a catalyst for connection, collaboration, and cultural change.

It offers a model for how pedagogical principles can be operationalized to support leadership development across levels and units.

It highlights the importance of continuity and organizational anchoring to sustain leadership practices over time.

Together, the findings suggest that leadership development is most effective when it is embedded in shared experiences, supported by competent facilitators, and guided by principles that foster trust, involvement, and recognition. The study underscores the value of designing leadership programs that move beyond individual competence to build collective capacity and organizational resilience.
